# Evaluating the use of a recommender system for selecting optimal messages for smoking cessation: patterns and effects of user-system engagement

**DOI:** 10.1186/s12889-021-11803-8

**Published:** 2021-09-26

**Authors:** Jinying Chen, Thomas K. Houston, Jamie M. Faro, Catherine S. Nagawa, Elizabeth A. Orvek, Amanda C. Blok, Jeroan J. Allison, Sharina D. Person, Bridget M. Smith, Rajani S. Sadasivam

**Affiliations:** 1 Division of Health Informatics and Implementation Science, Department of Population and Quantitative Health Sciences, University of Massachusetts Chan Medical School, 368 Plantation Street, Worcester, MA 01605 USA; 2 Department of Internal Medicine, Wake Forest School of Medicine, Winston-Salem, NC USA; 3 VA Center for Clinical Management Research, VA Ann Arbor Healthcare System, United States Department of Veterans Affairs, Ann Arbor, MI USA; 4 Department of Systems, Populations and Leadership, School of Nursing, University of Michigan, Ann Arbor, MI USA; 5 Division of Biostatistics and Health Services Research, Department of Population and Quantitative Health Sciences, University of Massachusetts Chan Medical School, Worcester, MA USA; 6 Center of Innovation for Complex Chronic Healthcare, Spinal Cord Injury Quality Enhancement Research Initiative, Hines VA Medical Center, Chicago, IL USA; 7 Department of Pediatrics, Feinberg School of Medicine, Northwestern University, Evanston, IL USA

**Keywords:** Digital health intervention, Smoking cessation, Computer-tailored health communication, Recommender system, Motivational messaging, Engagement

## Abstract

**Background:**

Motivational messaging is a frequently used digital intervention to promote positive health behavior changes, including smoking cessation. Typically, motivational messaging systems have not actively sought feedback on each message, preventing a closer examination of the user-system engagement. This study assessed the granular user-system engagement around a recommender system (a new system that actively sought user feedback on each message to improve message selection) for promoting smoking cessation and the impact of engagement on cessation outcome.

**Methods:**

We prospectively followed a cohort of current smokers enrolled to use the recommender system for 6 months. The system sent participants motivational messages to support smoking cessation every 3 days and used machine learning to incorporate user feedback (i.e., user’s rating on the perceived influence of each message, collected on a 5-point Likert scale with 1 indicating strong disagreement and 5 indicating strong agreement on perceiving the influence on quitting smoking) to improve the selection of the following message. We assessed user-system engagement by various metrics, including user response rate (i.e., the percent of times a user rated the messages) and the perceived influence of messages. We compared retention rates across different levels of user-system engagement and assessed the association between engagement and the 7-day point prevalence abstinence (missing outcome = smoking) by using multiple logistic regression.

**Results:**

We analyzed data from 731 participants (13% Black; 73% women). The user response rate was 0.24 (SD = 0.34) and user-perceived influence was 3.76 (SD = 0.84). The retention rate positively increased with the user response rate (trend test *P* < 0.001). Compared with non-response, six-month cessation increased with the levels of response rates: low response rate (odds ratio [OR] = 1.86, 95% confidence interval [CI]: 1.07–3.23), moderate response rate (OR = 2.30, 95% CI: 1.36–3.88), high response rate (OR = 2.69, 95% CI: 1.58–4.58). The association between perceived message influence and the outcome showed a similar pattern.

**Conclusions:**

High user-system engagement was positively associated with both high retention rate and smoking cessation, suggesting that investigation of methods to increase engagement may be crucial to increase the impact of the recommender system for smoking cessation.

**Trial registration:**

Registration Identifier: NCT03224520. Registration date: July 21, 2017.

**Supplementary Information:**

The online version contains supplementary material available at 10.1186/s12889-021-11803-8.

## Background

Effectiveness data for digital health or electronic health (eHealth) interventions to promote smoking cessation continues to grow [1–4]. Digital health interventions can provide several functions, including motivational messaging, facilitating connections with experts, and peer communities. Motivational messaging is a frequently used function in digital health interventions and has been adopted in several real-world smoking cessation programs [1–5]. Tailored motivational messaging, also called computer-tailored health communication (CTHC), is the process of selecting optimal motivational messages for an individual participant to improve the relevance of the messages [6]. CTHC builds on the concepts of personal relevance, relatedness, and cultural similarity [7–9]. It has been widely used to motivate behavior change, including smoking cessation [10–20]. CTHC systems can be implemented in many ways. Most CTHC systems are rule-based, where patients’ baseline characteristics (e.g., age, race, sex) are matched to if-then rules to select messages [6, 21]. One limitation of these systems is that they use pre-designed rules, and when being used, do not seek user feedback to improve message selection.

A new alternative approach to developing CTHC systems is using a recommender system [22–25]. The recommender system studied here is a system recently developed and applied to the domain of smoking cessation [22, 23]. The system actively seeks feedback from users (i.e., asking users to rate the motivational messages the system sent) and uses the feedback and other information about the users to inform the selection of the next message. The recommender system used a hybrid machine learning algorithm, which combined collaborative filtering and content-based ranking, to select messages that are most suitable for individual smokers [22, 23]. It used multiple information sources as its input to generate the recommendations (i.e., selecting the messages for users): metadata description of the messages, smokers’ demographic characteristics, and feedback data (e.g., smokers’ ratings on the messages). The recommender system had shown promising results in motivating smokers to quit in a pilot study [26]. This pilot study tested the short-term (30 days) effects of the recommender system on smokers (*N* = 120). During the 30-day follow-up, the intervention group (using the recommender system; *n* = 74) rated the message as influential (i.e., agreed or strongly agreed that the messages influenced them to quit smoking) more frequently than the comparison group (using the rule-based CTHC system; *n* = 46) (74% vs. 45%, *P* < 0.01). Among those who completed the follow-up, 36% (20/55) of intervention participants and 32% (11/34) of the comparison participants stopped smoking for 1 day or longer (*P* = 0.70).

The aim of the current study was to observe and analyze the behavior of the recommender system for promoting smoking cessation in a real-world setting to gain insights about how the system worked. We studied this from the perspective of user-system engagement and its impact on the cessation outcome. Our study was motivated by three reasons. First, it is usually assumed that digital health interventions need a certain level of user engagement to produce effects [27–29]. Second, measuring engagement is important for understanding the effects of digital health interventions on promoting behavior change [29, 30]. Third, traditional CTHC systems are one-way communication systems, which does not allow a closer examination of the user-system engagement. The recommender system, by design, seeks user feedback on the message it sent and uses this feedback to improve the selection of the next message. This system property provides us a unique opportunity to examine the granular user-system engagement patterns in terms of both behavior (e.g., user’s response rate) and subjective experience (e.g., user-perceived influence of the messages sent by the system) [28, 31].

In this report, we present our exploration of the following questions. How did the users engage with the recommender system? Did the recommender system perform and respond to users’ feedback as expected? What was the impact of user-system engagement on retention? What was the association between user-system engagement and the smoking cessation outcome?

## Methods

### Study overview

We prospectively followed a cohort of current smokers enrolled in the Smoker2Smoker study [32] which aimed to use a recommender system to promote smoking cessation, including encouraging access to an online health communication program for tobacco cessation [33]. During the 6-month intervention period, the recommender system sent motivational messages to participants every 3 days, and encouraged the participants to rate each message (see Additional File 1 for an example). The message pool contained 500 motivational messages developed in our prior study, which included both theory-driven, expert-written messages and peer-written messages [34]. The system used a machine learning algorithm that combined content-based ranking and collaborative filtering methods to select the next message for each individual participant. The algorithm predicted the rating of each message for each user by using (1) user-based and message-based features and (2) the information extracted from the rating-score matrix for all the users and messages [22, 23]. Participant’s retention and 7-day point prevalence abstinence were assessed at their 6-month follow-up.

To answer the questions described previously (see the Background section), we first examined the user-system engagement using various metrics, measuring either users’ response and feedback for the system or the system’s response to user feedback. We then assessed the impact of user-system engagement (measured by user response rate and user-perceived message influence) on retention and the 7-day point prevalence abstinence.

### Sample of current smokers

English-speaking, current smokers aged ≥18 years were recruited nationally between July 2017 and March 2019. Two methods were used to recruit participants [32]. The primary method was to directly recruit participants online using advertisements posted through search engines, social media and smokefree.gov or using ResearchMatch. The secondary method was to recruit participants through their friends and family members who had participated in the program and had access to the peer recruitment tools. The protocol of this study was approved by the Institutional Review Board at the University of Massachusetts Chan Medical School. All participants provided written informed consent for study participation.

### Data collection

#### Baseline characteristics

Participants completed a baseline survey at registration or within 1 week after registration, which collected participants’ demographics, socio-economic status, and other background information.

#### Users’ response to the recommender system and ratings for messages

During the 6-month intervention, we collected each participant’s (i.e., user’s) ratings on the messages sent by the recommender system. Participants were asked to rate the influence of each message that they received by answering a 5-point Likert scale question, “Does this message influence you to quit smoking?” with response choice of strongly disagree, disagree, neutral, agree, strongly agree (corresponding to scores 1 to 5). Participants were not incentivized for rating the messages, allowing for heterogeneity in levels of engagement with the system. We computed measures for user-system engagement using these data, as detailed in the Data Analyses section.

#### Outcomes

Participants were asked to complete a survey at their 6-month follow-up, which included one question: “Do you currently smoke cigarettes (smoked even 1 puff in the last 7 days)?” to collect self-reported 7-day point prevalence abstinence [35, 36].

The survey was administered online (the primary delivery mode) or through phone calls. We identified dropouts (i.e., lost to follow-up) in the following way. The research team sent each participant up to 4 email reminders to complete the online 6-month follow-up survey within 2 weeks following the targeted follow-up date. Participants who did not respond to email reminders were called to complete the survey over the phone. Participants who failed to complete the outcome survey online or by phone were treated as dropouts.

### Data analyses

We first compared baseline characteristics across 4 levels of participant’s engagement with the system using the chi-square test. We defined the 4 levels of engagement by using participants’ response rates (i.e., the number of messages rated by a participant divided by the number of messages the participant received). We then explored our research questions with the methods described in the following subsections.

#### User engagement with the recommender system

To assess how the users (i.e., participants) engaged with the recommender system, we characterized a user’s engagement behavior by several measures based on the user’s responses. We first defined the user’s response rate as the number of messages rated divided by the number of messages received. We then defined four levels of response rate, with similar number of users assigned to each level. Specifically, we assigned zero response rates into the first level. We then identified the tertile values for the non-zero response rates. We used the values (accurate to one decimal place) closest to these tertile values to divide the non-zero response rates into three levels: low response: > 0 & ≤0.1, medium response: > 0.1 & ≤0.6, and high response: > 0.6 & ≤1. Other measures included whether the user responded to the first message, whether the user responded to the last message, and whether the user withdrew from the message sending list (i.e., declined to receive more motivational messages) during the intervention. We reported the number and percent of users for subgroups categorized by these measures.

In addition to measuring user engagement for the whole 6-month period, we examined the trend of user engagement over time using the following method. We divided the 6-month follow-up period into 6 timespans. For each timespan or month, we first calculated each user’s response rate and then calculated two metrics at the population level. The first metric is the mean of the users’ response rates during a timespan. The second metric is the percent of users who did not respond to any messages during a timespan. We reported user engagement over time measured by these two metrics for three levels of 6-month response rate, i.e., low, medium, and high responses, as defined previously.

#### The recommender system’s performance and response to user feedback

We developed three methods to assess whether the recommender system performed or responded to user feedback as expected, using user’s rating about the influence of the messages.

We first defined the rating score as a numeric value (between 1 and 5) associated with a user’s response to the 5-point Likert scale question “Does this message influence you to quit smoking?” (with 1 for “strongly disagree” and 5 for “strongly agree”).

In the first method, for each user, we averaged their rating scores for the messages they received, resulting a single rating score per user. Using these user-level rating scores, we estimated the recommender system’s performance by: (1) averaging the rating scores of all the users; (2) categorizing users into subgroups based on their rating scores and calculating user distribution across these subgroups.

In the second method, for each user, we calculated the percent of times the user rated the messages as influential (i.e., “agree” or “strongly agree” in response to the influence question). We then categorized users into subgroups based on this information and calculated user distribution across the subgroups.

In the third method, we assessed the system’s response to user’s negative feedback in the following way. First, for each user, we calculated the percent of times that, when the system received a low rating (“strongly disagree”, “disagree”, or “neutral”) from this user, it was able to send the user the next message with a higher rating score. We then assigned the users to different subgroups based on this information and calculated user distribution across the subgroups. For example, if the system was able to improve the next message 80% or more times for a user when receiving a low rating score from this user, we will assign this user into one subgroup. The more users fall into this subgroup, the better the system performs in responding to negative feedback.

Note that metrics defined by the first two methods also measure user engagement with the system. Different from the metrics described in the previous section, these metrics are based on subjective experience, i.e., user perceptions of how much the messages sent by the system had influenced them to quit smoking.

#### Impact of demographic factors on user-system engagement

As an additional analysis, we assessed the impact of demographic factors on user-system engagement. Specifically, we calculated and compared the mean response rate and the mean rating score of perceived message influence across subgroups categorized by the demographic factors. For each factor, we used ANOVA to test its overall effects on the response rate and the rating score respectively. For each polytomous factor that had an overall significant effect on the response rate or the rating score, we used the Bonferroni multiple-comparison test to assess the difference between each pair of categories.

#### High-impact motivational messages

We also conducted an analysis to identify motivational messages that may have had the most impact on users. Because the rating score of a message reflected user-perceived influence of the message on quitting smoking, we identified high-impact messages using the mean rating score each message received. We included only messages that had been sent to at least 20 users to improve the reliability of the estimates of means. We then identified the top-10 messages that had the highest mean rating scores and identified 1 or 2 content focuses of these messages.

#### Engagement and retention

To assess the impact of user-system engagement on retention, we calculated the retention rate (1 - dropout rate) at 6-month follow-up across the levels of user response rate and the levels of user’s influence rating.

#### Engagement and smoking cessation

To assess the impact of user-system engagement on smoking cessation, we examined the association between engagement and the 7-day point prevalence abstinence at 6-month follow-up.

In the previous subsections, we defined two types of engagement. The first one focuses on user response rate and the second one focuses on the system’s performance and response to feedback (as reflected by user’s perceived influence of the messages). Because variables of the same type are correlated with each other except for the variable representing user’s unsubscribing to the system, we chose one variable for each type, i.e., the level of user response rate and the level of perceived influence measured by the rating score, for the association analyses.

Intuitively, the level of response rate and the level of perceived influence can be also related in a scenario where the users are more likely to respond to the messages when they think the messages are helpful or influential. The bivariate analysis showed that these two variables did correlate but not in a simple, linear pattern. For this reason, we assessed the association between each of them and the 7-day point prevalence abstinence respectively.

In the association analysis, we treated dropouts and participants lost to follow-up (i.e., participants missing cessation outcome) as smoking [37]. This method is commonly used in smoking cessation studies [1, 38]. As the rating score of perceived influence missed values for over 1/3 participants who did not respond to the system, we treated the missed values as a single value “no rating” when analyzing the association between the rating score and the cessation outcome. We used multivariable logistic regression to adjust for potential confounders that were identified from statistical analysis (Table 1, *P* < 0.05). In addition, we adjusted for two factors: the number of messages a participant received and whether the participant unsubscribed from receiving messages from the system during the 6-month intervention period.

**Table 1:** Participant characteristics at baseline, by level of engagement^a^

	Levels of engagement (response rate)				***P*** value^**b**^
	0	> 0 & ≤0.1	> 0.1 & ≤0.6	> 0.6 & ≤1	
	***n*** = 279	***n*** = 153	***n*** = 151	***n*** = 148	
	n (%)	n (%)	n (%)	n (%)	
Age group					< 0.001*
19–24 years	29 (10.4)	8 (5.2)	16 (10.6)	6 (4.1)	
25–34 years	52 (10.4)	32 (20.9)	41 (27.2)	22 (14.9)	
35–44 years	44 (18.6)	18 (11.8)	34 (22.5)	41 (27.7)	
45–54 years	44 (15.8)	30 (19.6)	16 (10.6)	30 (20.3)	
55–64 years	82 (29.4)	50 (32.7)	34 (22.5)	41 (27.7)	
65+ years	28 (10.0)	15 (9.8)	10 (6.6)	8 (5.4)	
Gender					0.24
Female	200 (71.7)	120 (78.4)	103 (68.2)	108 (73.0)	
Male	79 (28.3)	33 (21.6)	48 (31.8)	40 (27.0)	
African-American					0.01*
Yes	35 (12.5)	11 (7.2)	30 (19.9)	21 (14.2)	
No	244 (87.5)	142 (92.8)	121 (80.1)	127 (85.8)	
Education					0.35
≤ High school	37 (28.0)	29 (32.6)	34 (25.8)	40 (28.0)	
Some college or technical school	62 (47.0)	36 (40.4)	64 (48.5)	53 (37.1)	
College graduate	33 (25.0)	24 (27.0)	34 (25.8)	50 (35.0)	
How hard it is for you/family to pay for medical care					0.27
Very hard	23 (17.4)	17 (19.1)	20 (15.0)	33 (23.1)	
Hard	17 (12.9)	17 (19.1)	21 (15.8)	20 (14.0)	
Somewhat hard	49 (37.1)	27 (30.3)	44 (33.1)	43 (30.1)	
Not very hard	40 (30.3)	28 (31.5)	40 (30.1)	45 (31.5)	
Don’t know	3 (2.3)	0 (0.0)	8 (6.0)	2 (1.4)	
Number of cigarettes smoked per day					0.001*
< =10	84 (30.1)	47 (30.7)	74 (49.0)	49 (33.1)	
> 10 and < =20	132 (47.3)	78 (51.0)	59 (39.1)	62 (41.9)	
> 20	63 (22.6)	28 (18.3)	18 (11.9)	37 (25.0)	

* indicates statistically significant (*P* < 0.05)

^a^Baseline characteristics of 731 participants by four levels of engagement (response rate = 0, > 0 and ≤ 0.1, > 0.1 and ≤ 0.6, > 0.6 and ≤ 1.0)

^b^We used chi-square test to assess the difference in user engagement levels over categorical variables

Statistical analyses were performed using STATA/IC 15.1 [39].

## Results

Among the 731 participants analyzed in this study, 59 (8%) were under 25 years of age and 268 (37%) were over 54 years of age; 97 (13%) were Black; and 531 (73%) were women. Age, race, and the number of cigarettes smoked per day before participating the study were significantly different across the levels of the participant’s engagement (i.e., user response rate) with the system (Table 1).

### User engagement with the recommender system

User’s engagement with the recommender system was heterogenous (Table 2). Among the 731 users, 148 (20%) responded to over 60% messages they received and 279 (38%) did not respond to any messages. Most users did not respond to the first message or the last message they received. Over 15% of the users unsubscribed to receiving messages during the 6-month intervention period.

**Table 2:** Engagement with the recommender system among 731 users

	n or mean [std]	Percent, %
overall response rate (mean [SD])^a^	24% [34%]	
levels of response rate		
0	279	38.2
> 0 & ≤0.1	153	20.9
> 0.1 & ≤0.6	151	20.7
> 0.6 & ≤1	148	20.2
did not respond to the first message	428	58.6
did not respond to the last message	611	83.6
unsubscribed to receiving messages	113	15.5

^a^The response rate for each user is defined as the number of messages the user rated by the total number of messages the user received. On average, a user received 59 (SD = 16) messages and responded to 13 (SD = 20) messages. The overall response rate was calculated by averaging the response rates of the 731 users

**Fig. 1:**
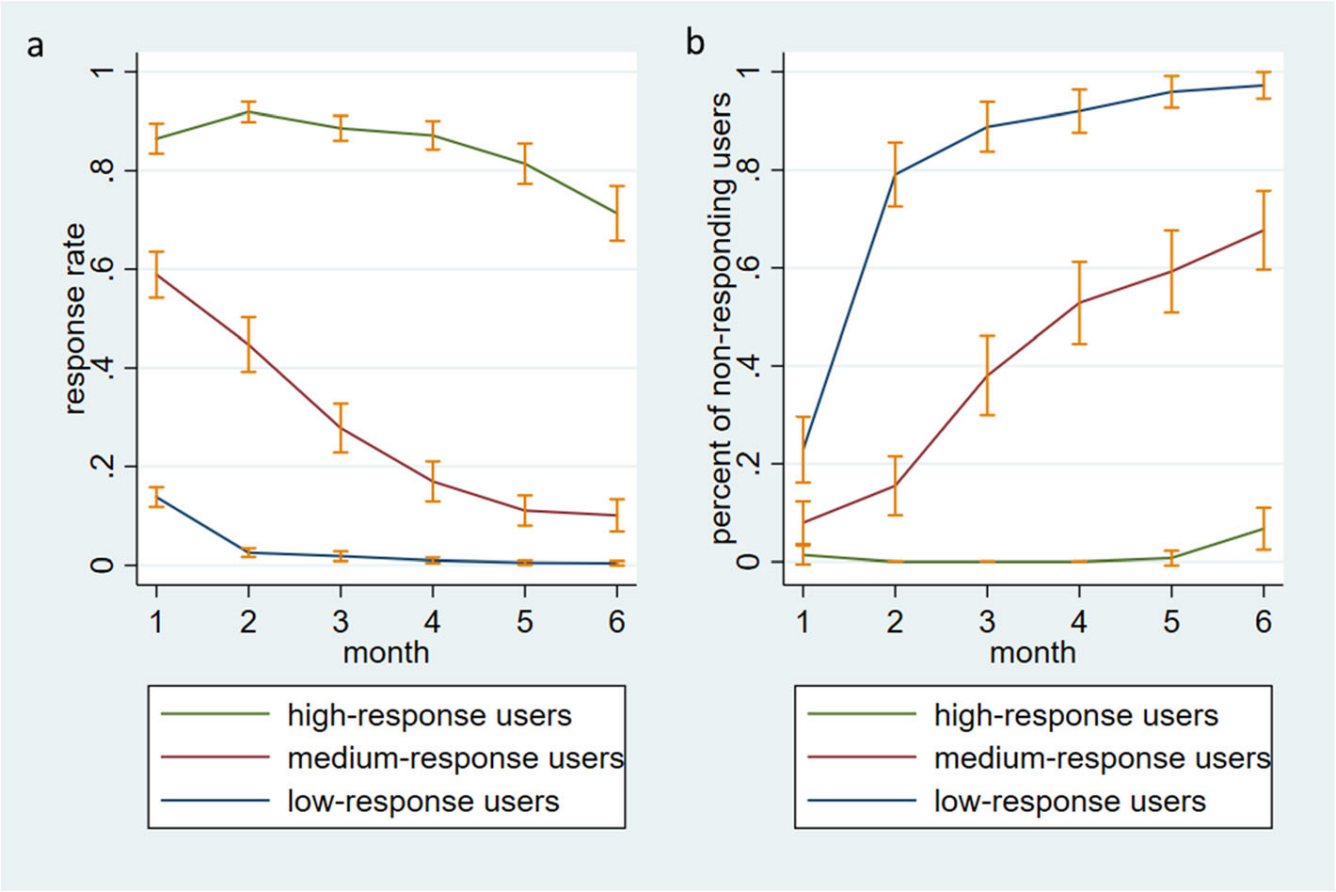
Trend of responses over time, for users with different levels of 6-month response rate. High-response rate: > 0.6 & ≤1, Medium-response rate: > 0.1 & ≤0.6, Low-response rate: > 0 & ≤0.1

User engagement over time differed between highly-engaged and low-engaged users (Fig. 1; see details in Additional File 2). For example, the mean response rate of the low-response users dropped substantially from the 2nd month while the response rate of high-response users did not drop much until the 5th month (Fig. 1a). The descending trend of the response rate of the medium-response users was smoother, compared with two other groups (Fig. 1a). The difference in the percent of users not responding to any messages per month showed similar patterns (Fig. 1b).

### The recommender system’s performance and response to user feedback

Among the 452 users who have rated messages from the recommender system, the average rating score of perceived message influence was 3.76 (Table 3). Only 12% of these users rated the messages as not influential (i.e., the user’s average rating score ≤ 3.0); 36% rated the messages very high (i.e., the user’s average rating score > 4.5); 37% rated all the messages they have received as influential (i.e., rating score > 3).

**Table 3:** Recommender system’s performance rated by users (*n* = 452) and its response to user feedback^a^

	n or mean [std]	Percent, %
**User-perceived influence of motivational messages**		
Rating score	3.76 [0.84]	
Level of average rating scores (“Does this message influence you to quit smoking”)		
≤ 3.0 (from strongly disagree to neutral)	55	12.2
> 3.0 & ≤4.0 (agree)	138	30.5
> 4.0 & ≤4.5 (strongly agree, level I)	96	21.2
> 4.5 & ≤5 (strongly agree, level II)	163	36.1
Percent of times when a user rated a message as influential^b^		
< 0.5	72	15.9
≥ 0.5 & < 0.8	93	20.6
≥ 0.8 & < 1.0	119	26.3
1.0	168	37.2
**Response to user’s negative feedback**		
After receiving a low score (i.e., rating score ≤ 3) from a user, percent of times the next message received a higher score, calculated for each user^c^		
< 0.5	25	10.2
≥ 0.5 & < 0.8	78	31.7
≥ 0.8	143	58.1

^a^452 (among 731) users rated at least one message they received

^b^An influence rating score > 3 (i.e., strongly agree or agree to the question “Does this message influence you to quit smoking”) was regarded influential

^c^This analysis was conducted for users (*n* = 246) who had rated at least one message they received as non-influential (rating score ≤ 3) and also rated the next message the system sent. For each of the 246 users, we calculated the percent of times that, when the recommender system received a low score (≤ 3) from the user on the current message, it was able to receive a higher score (i.e., improved the quality) for the next message it sent

There were 246 users who had rated at least one message they received as non-influential (rating score ≤ 3) and also rated the next message the system sent. For 143 (58%) of these users, the system was able to improve the quality of the next message over 80% of the time when receiving a low score (≤ 3) on the current message (the last row in Table 3).

### Impact of demographic factors on user-system engagement

User-system engagement was impacted by demographic factors. For example, users with age of 35–44 years had a higher response rate, compared with users with age of 19–24 (18.8 vs. 8.4, *P* = 0.01) and 25–34 (18.8 vs. 11.4, *P* = 0.03) years. African-American users assigned higher influence rating scores to the messages than non-African-American users (4.3 vs. 4.1, *P* = 0.03). College graduates assigned lower rating scores than users with high school or lower-level education (4.0 vs. 4.2, *P* = 0.03) and users with some college or technical school education (4.0 vs. 4.2, *P* = 0.04). The detailed results were summarized in Additional File 3.

### High-impact motivational messages

Using users’ rating scores of perceived influences, we identified 10 messages that had the most impact (see Additional File 4). Each of these messages focuses on 1 or 2 topics. Specifically, 6 messages focus on treatment methods (5 on behavior treatments and 1 on both general and behavior treatments), 4 focus on benefits of quitting smoking (2 on health benefits and 2 on financial benefits), and 2 focus on motivation to quit.

### Engagement and retention

**Fig. 2:**
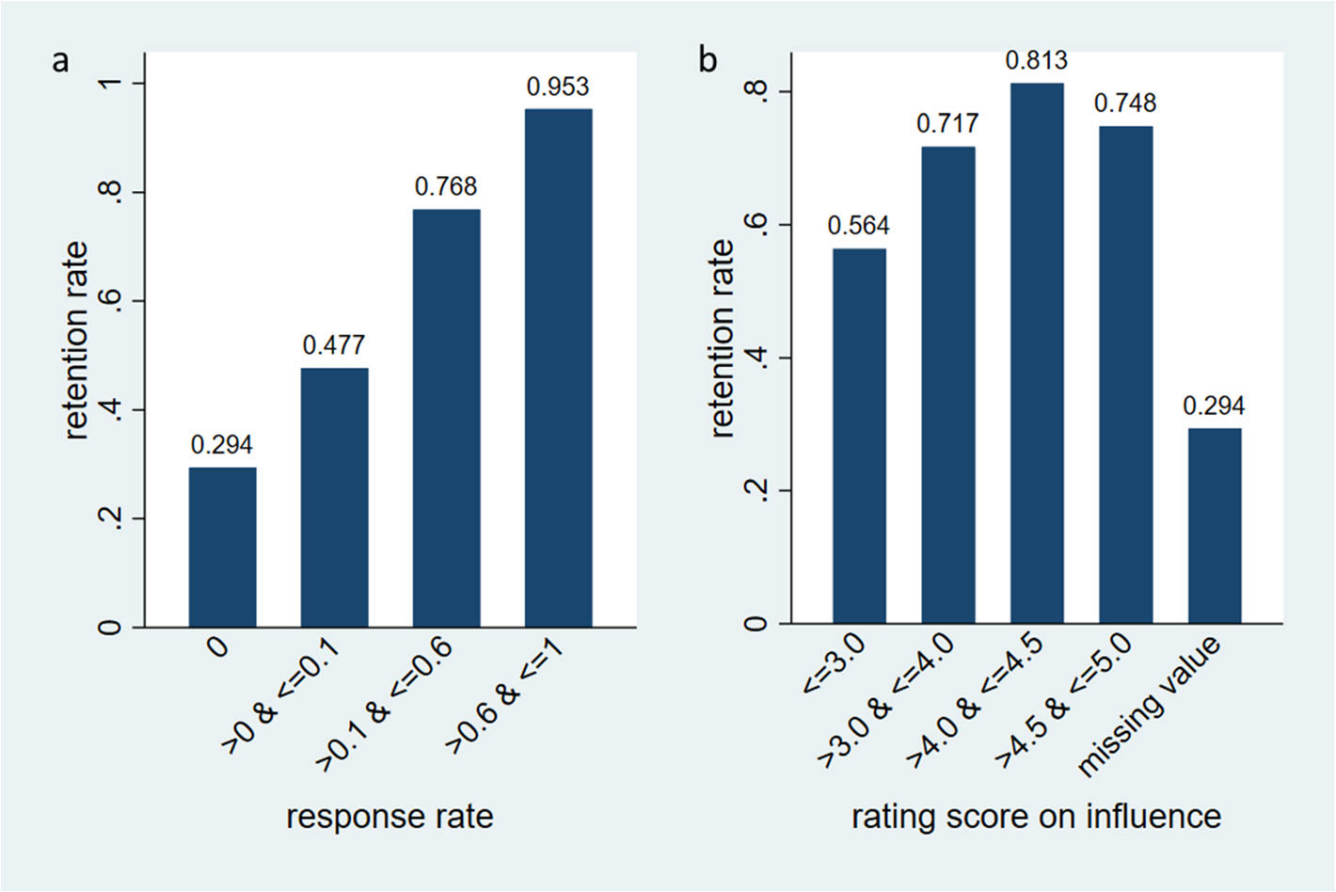
Retention rate by level of (**a**) response rate (*P* < 0.001) and (**b**) rating score of perceived influence

The overall retention rate was 0.56. The retention rate positively increased with user response rate (Fig. 2a, trend test *P* < 0.001). It also positively increased with the rating score of perceived influence of messages, but the trend stopped when the rating score was higher than 4.5 (Fig. 2b).

### Engagement and smoking cessation

**Fig. 3:**
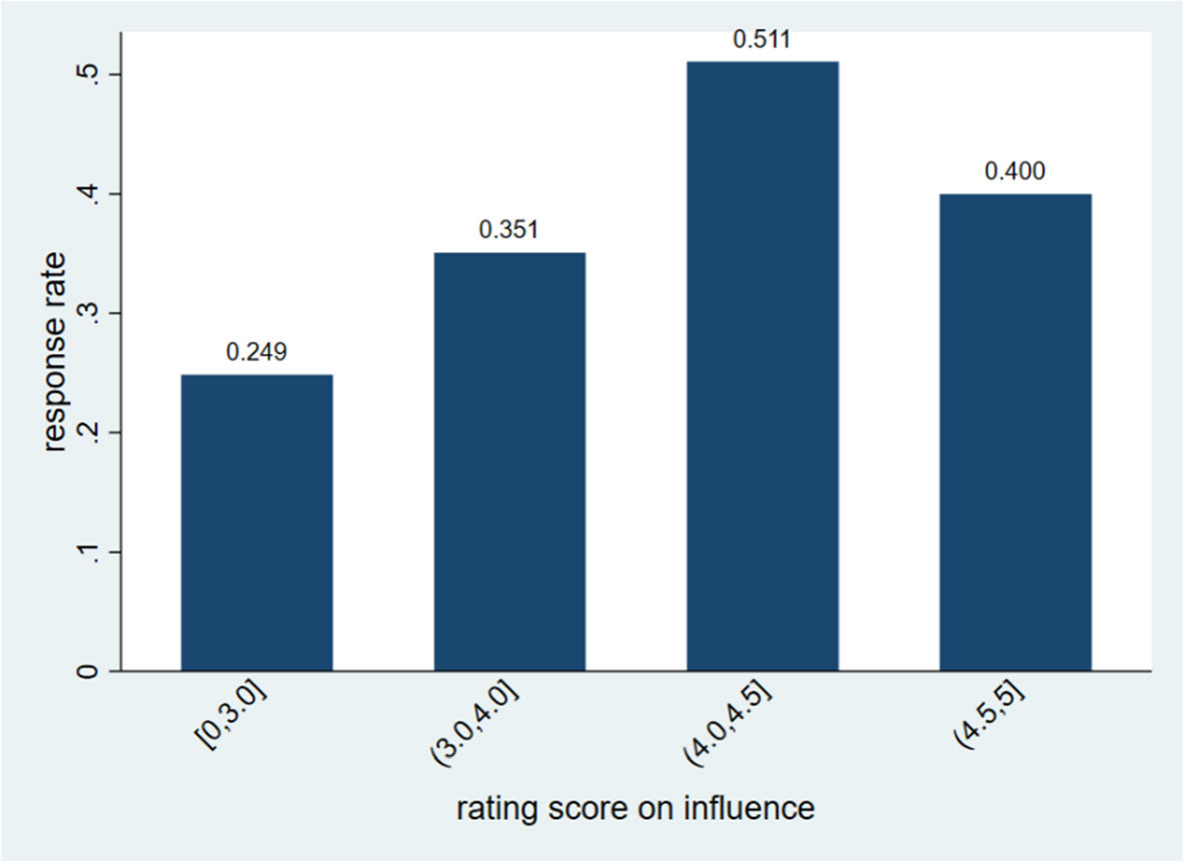
Response rate by level of rating score of perceived influence (*P* < 0.001)

The level of a user’s response rate was correlated with the user’s rating score of message influence (*P* < 0.001). However, the response rate did not increase all the way through when the rating score increased. Instead, it leveled out after the rating score reached 4.5 (Fig. 3).

There was a trend of increased cessation rate from non-response, low response rate (≤0.1), moderate response rate (> 0.1 and ≤ 0.6), to high response rate (> 0.6) (12.5% vs. 19.6% vs. 27.2 vs. 27.0). The trend was more obvious after adjusting for potential confounders (Model 2 in Table 4). In particular, compared with non-response, the odds of cessation outcome increased from low response rate (odds ratio [OR] = 1.86, 95% confidence interval [CI]: 1.07–3.23), moderate response rate (OR = 2.30, 95% CI: 1.36–3.88), to high response rate (OR = 2.69, 95% CI: 1.58–4.58).

**Table 4:** Association between engagement and self-reported 7-day point prevalence abstinence (missing = smoking) at 6 months

	Incidence of Cessation	Model 1: Unadjusted		Model 2: Adjusted^a^	
	n/N (%)	OR (95% CI)	*P* value	OR (95% CI)	*P* value
**Response rate**					
Non-response (0)	35/279 (12.5)	Reference		Reference	
Low (> 0 & ≤0.1)	30/153 (19.6)	1.70 (1.00, 2.90)	0.05	1.86 (1.07, 3.23)	0.03*
Moderate (> 0.1 & ≤0.6)	41/151 (27.2)	2.60 (1.57, 4.30)	< 0.001*	2.30 (1.36, 3.88)	0.002*
High (> 0.6 & ≤1.0)	40/148 (27.0)	2.58 (1.56, 4.29)	< 0.001*	2.69 (1.58, 4.58)	< 0.001*
**Message influence rating score**					
Low (≤3.0)	6/55 (10.9)	Reference		Reference	
Moderate (> 3.0 & ≤4.0)	28/138 (20.3)	2.08 (0.81, 5.34)	0.1	2.14 (0.83, 5.51)	0.1
Good (> 4.0 & ≤4.5)	24/96 (25.0)	2.72 (1.04, 7.15)	0.04*	2.76 (1.04, 7.30)	0.04*
Excellent (> 4.5 & ≤5)	53/163 (32.5)	3.93 (1.59, 9.76)	0.003*	4.04 (1.62, 10.10)	0.003*
No rating	35/279 (12.5)	1.17 (0.47, 2.94)	0.7	1.21 (0.48, 3.05)	0.7

* indicates statistically significant (*P* < 0.05)

^a^Model 2 for response rate was adjusted by age, race, and daily cigarettes assessed at baseline, whether the user unsubscribed to the recommender system, and the number of messages received by the user during the 6 months. Model 2 for message influence rating score was adjusted by race, whether the user unsubscribed to the recommender system, and the number of messages received by the user during the 6 months

Similarly, there was a trend of increased cessation rate from low rating (≤ 3.0), moderate rating (> 3.0 and ≤ 4.0), good rating (> 4.0 and ≤ 4.5), to excellent rating (> 4.5) (10.9% vs. 20.3% vs. 25.0 vs. 32.5). Compared with low rating, the odds of cessation outcome increased from moderate rating (OR = 2.14, 95% CI: 0.83–5.51), good rating (OR = 2.76, 95% CI: 1.04–7.30), to excellent rating (OR = 4.04, 95% CI: 1.62–10.10) after adjusting for covariates.

## Discussion

Tailored motivational messaging or CTHC systems have proven effective in promoting smoking cessation [15–20], but the knowledge about smokers’ engagement with such systems is still limited. In this study, leveraging data around the use of a recommender system for CTHC, we examined the granular user-system engagement patterns during the intervention process and their impact on the cessation outcome. We found that user response rates for the recommender system were heterogenous, with 20% users responding to (i.e., rated) over 60% messages they received and 38% users not responding to any messages. Most users who responded to the system thought that the messages were influential. User’s response rate and perceived influence of the messages were correlated, but not in a simple linear pattern. Users with high response rates or giving the messages high influence rating scores were more likely to quit smoking at 6-month follow-up. Below we further discuss our findings within the context and their implications for future research.

Prior tailored motivational messaging systems typically used rule-based algorithms to select messages [15–20, 40–44], where user’s baseline characteristics are matched to if-then rules [6, 21]. In a prior study, we had implemented a rule-based system to select messages for a smoker based on their readiness to quit [17]. The Text2Stop system is another example of this rule-based approach. The system used user’s demographic and other information (such as smoker’s concerns about weight gain after quitting) collected at baseline to select motivational messages for each user [19]. The recommender system we studied is different from these previous systems. It proactively sought a user’s feedback (i.e., rating) on a message it sent and used the feedback to improve the selection of the next message for this user using a machine learning algorithm [22, 23]. The actions of seeking feedback, improving message selection, and sending the next message would continue if the user kept engaging with the system. In this study, leveraging this unique system property, we closely tracked and examined user-system engagement patterns and studied their impact on the cessation outcome.

Measuring engagement is important for understanding the effects of digital health interventions to promote behavior change [29, 30]. Previous work conceptualized engagement with digital behavior change interventions in terms of both behavior (e.g., extent of usage and reactions to the intervention) and subjective user experience (e.g., attention, interest, and affect) [28, 31]. A variety of methods, such as semi-structured interviews, observations, self-report questionnaires, ecological momentary assessment, psychophysiological measures, and the analysis of system usage data, have been applied to measure engagement [28, 30, 31]. Our method is closely related to ecological momentary assessment (EMA), which assesses users’ current experiences and behaviors in real time and in their natural environment [45]. The use of EMA in eHealth research has been focused on assessing health behavior and determinants rather than engagement [30]. Our study contributes to the literature by providing a small, successful use case of EMA-like methods in measuring engagement for eHealth interventions. For example, by examining the continuously tracked engagement data, we found that the low-engaged users tended to stop responding to the system much earlier than the highly-engaged users (Fig. 1).

To the best of our knowledge, our work was the first to measure user-system engagement around using a recommender system for smoking cessation. As there were no standard measures available for this problem, our measure design was guided by general principles recommended in the engagement literature and also driven by our data. For example, we defined two types of measures for user-system engagement, as represented respectively by user’s response rate and user’s rating score for the influence of the messages sent by the system. This was informed by the literature on measuring engagement for digital health interventions, which emphasized the importance of measuring both behavior and user experience [28, 31]. Our two measures complement each other in several ways. The user response rate is an objective measure that focuses on user behavior; the influence rating score is a subjective measure that focuses on user experience and perceptions. The former quantifies the engagement for all users receiving the motivational messages; while the latter focuses on users who have responded to the motivational messages. Intuitively, we expect users to be more willing to respond to the system if they think the messages sent by the system are influential. In our study, we found that these two measures were indeed correlated, but their relationship was not a simple, linear one (Fig. 3). This suggests that the two measures are different and both are valuable for measuring engagement.

When assessing user engagement by the response rate, we used both the mean response rate and four levels of response rate. The second measure provided additional information about user engagement when the user response rates were not normally distributed (as in our case). We used a data-driven approach to define this measure (see Data Analyses) because there were no standard criteria for defining the level of engagement. Therefore, the numeric values we used to define the levels of engagement may not be generalizable to other studies. However, our data-driven approach, including separating zero-response users from other users and using the quantiles to inform the definition of response levels, is generic and can be applied to other study settings.

Light-touch digital health interventions like motivational messaging are more accessible to study participants than in-person or telephone counseling, and therefore are likely to reach a wider population of smokers. However, low levels of engagement and high rates of drop-out are common in such interventions [46–48], which may reduce their impact. In this study, we found a retention rate (i.e., 1 – dropout rate) of 0.56 for using the recommender system. This level of retention rate is low, and is comparable to several prior studies using digital interventions, including motivational messaging, to promote smoking cessation [17, 18, 20, 40, 49, 50]. In addition, we found a high-degree of heterogeneity in the user-system engagement measured by user’s response rate. Active users (20%) responded to over 60% messages they received; while quite some users (38%) did not respond to the system at all. The retention rate for highly-engaged users (> 0.6 response rate) was more than 3 times of the retention rate of zero-response users (0.953 vs. 0.294; Fig. 2a). A higher-level of engagement was also associated with a higher likelihood of quitting smoking (Table 4). These results, taken together, suggest that developing strategies to engage low-engaged users is the key to improve retention and the impact of the recommender system on promoting smoking cessation.

There are several strategies that may be useful for improving user’s engagement with the system. Our trend analysis of engagement showed that low-response users decreased their engagement with the system early (after 1 month of participation) (Fig. 1). We also found that low response rate was associated with low retention rate (Fig. 2a). In particular, the zero-response users had a very high dropout rate (0.706). These results suggest that having a strategy to track engagement and enhance the dose of intervention (e.g., having a tobacco cessation specialist to intervene) for low-response, and especially zero-response, participants at the early phase (e.g., the first couple of weeks) may improve engagement and prevent dropouts. In addition, our analysis results highlight improvements that can be made to the system. The protocol of the current recommender system is to send a user the same message next time if the user did not rate the message. This design can be suboptimal, especially when the user did not rate the message mainly because they did not like the message or think it helpful. In the future, we will explore the following strategies: (1) improving the next message selection by treating non-response lasting for more than 1 week as negative feedback; (2) randomly selecting another message to send; and (3) developing new methods to seek implicit feedback from users’ response patterns. Furthermore, strategies targeting young adults may be also useful. Prior studies found that young adults were less likely to use tobacco cessation treatment [51, 52], including web-based cessation interventions [53, 54]. Similarly, we found that younger adults were less likely to engage with the recommender system than older adults.

Because the rating score directly measures a user’s perceived influence of the motivational messages, it provides more information about the quality of the engagement than the response rate does. In this study, we found that, among users who have responded to the recommender system, 57% of them strongly agreed (i.e., average rating score > 4.0) and 37% always agreed that the messages they received were influential (Table 3). Among the top-10 high-impact messages ranked by the rating score, 6 messages focus on behavior treatment methods, indicating the important role of behavior therapy in smoking cessation. In addition, for 58% of the users, the system was able to improve its selection of the next message for over 80% cases where it received a low rating score (Table 3). These results indicate that the recommender system worked quite well for this subset of users. Compared with other users, African-American users assigned higher rating scores to the messages sent by the recommender system. This finding is compatible with that from our pilot study [55], suggesting that the recommender system may be helpful in engaging and motivating this harder-to-reach and harder-to-engage group. This hypothesis needs to be further tested in studies specifically targeting this group. Additionally, we found that the rating scores assigned by users were usually high and were very close to each other. Therefore, the amount of information that the system can learn from these scores may be limited. Using a wider scale (e.g., 1–10) for the rating score may be helpful in addressing this limitation.

Our study has several limitations. First, similar to other observational studies, we can’t ascertain causality. Although we have adjusted for baseline participant characteristics for the association analyses, there may be unobserved factors to impact the analysis results. Second, our messaging system is also limited in that there is a potential that participants may not have received the motivational messages we sent them. We were unable to confirm whether the zero-response cases were partially due to the failure in message delivery. Third, due to an issue with system setup at the beginning of this study, participants (less than 5%) who registered for the study before November 10, 2017 received daily messages before that date. To reduce the impact of this issue on data analysis, we used the response rate (rather than the total number of messages rated by a participant) to measure participant’s engagement with the system and adjusted the association analyses by the total number of messages received by each participant. Fourth, we found that user engagement leveled out after the rating score reached 4.5. However, it is hard to interpret this result due to lack of qualitative data from the users. Future studies that use both quantitative and qualitative methods to study the engagement are warranted to better understand this phenomenon. Finally, we had a low retention rate of 0.56 which was associated with low engagement, but we do not know specifically why these participants dropped out.

## Conclusions

This study assessed the granular user-system engagement around a recommender system that sent tailored motivational messages to individual smokers to promote smoking cessation. The data-driven approach we used to measure the level of engagement is generic and can be applied to other study setttings. In this study, we found that a high-degree of heterogeneity of user-system engagement existed. High engagement was positively associated with high retention rate and the smoking cessation outcome. These results suggest that improving user-system engagement is the key to increase the impact of the system. Strategies that intervene on lower-engaged users at an early stage may be helpful. Innovative methods to collect implicit feedback from the users can be also useful for improving the system performance. We are evaluating this recommender system in a pragmatic trial [32], and will be able to measure the overall impact of the recommender system, and also incorporate level of engagement as the pathway through which the system creates its effect.

## Supplementary Information

**Additional file 1.** Interaction between a user and the recommender system that motivates smoking cessation.

**Additional file 2.** Trend of user-system engagement over time.

**Additional file 3.** Impact of demographic factors on user-system engagement.

**Additional file 4.** Top-10 high-impact messages sent by the recommender system.

## Data Availability

The data underlying this article will be shared on reasonable request (for non-commercial research) to the Principal Investigator of the project (Rajani.Sadasivam@umassmed.edu) and through a data-sharing agreement.

## Abbreviations

CI: Confidence interval
CTHC: Computer-tailored health communication
eHealth: Electronic health
EMA: Ecological momentary assessment
OR: Odds ratio
