# Supplementary material for: Evaluating the use of a recommender system for selecting optimal messages for smoking cessation: patterns and effects of user-system engagement
Source: BMC Public Health. 2021 Sep 26;21:1749. doi: 10.1186/s12889-021-11803-8 (PMC8465689; doi:10.1186/s12889-021-11803-8)

# Additional File 1: Interaction between a user and the recommender system that motivates smoking cessation

Step 1: The user received a tailored email message from the recommender system to motivate quitting smoking and a request to rate the influence of the message on him/her.

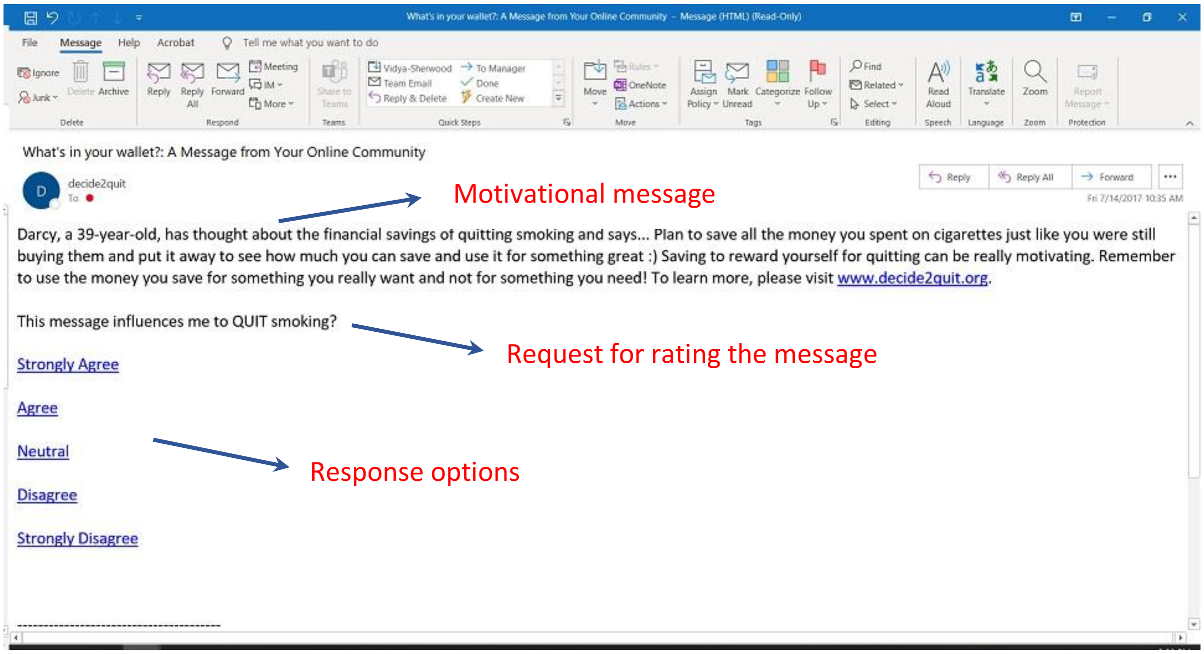


Step 2: After the user rated the message by choosing/hitting one of the response options, their answer was automatically registered into the database of the recommender system and the system showed a new webpage (see the figure below) to acknowledge the receipt of the user's response/rating.

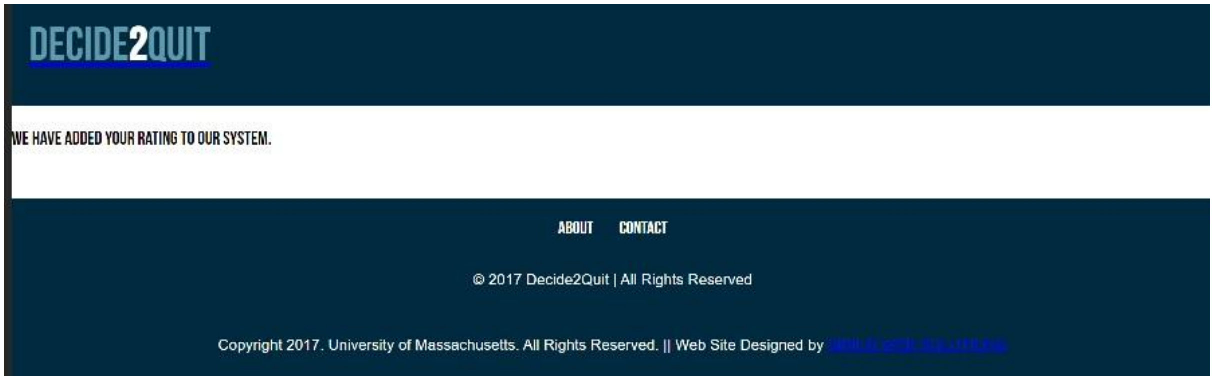

Supplement: Supplementary file 1 — Additional file 1. Interaction between a user and the recommender system that motivates smoking cessation. [file 12889_2021_11803_MOESM1_ESM.pdf]
