# Supplementary material for: Evaluating the use of a recommender system for selecting optimal messages for smoking cessation: patterns and effects of user-system engagement
Source: BMC Public Health. 2021 Sep 26;21:1749. doi: 10.1186/s12889-021-11803-8 (PMC8465689; doi:10.1186/s12889-021-11803-8)
Supplement: Supplementary file 2 — Additional file 2. Trend of user-system engagement over time. [file 12889_2021_11803_MOESM2_ESM.pdf]

Additional File 2: Trend of user-system engagement over time

The following figure provides detailed information about the data points in Figure 1 from the main paper.

Figure A2-1. Trend of responses over time, for users with different levels of 6-month response rate

(a)

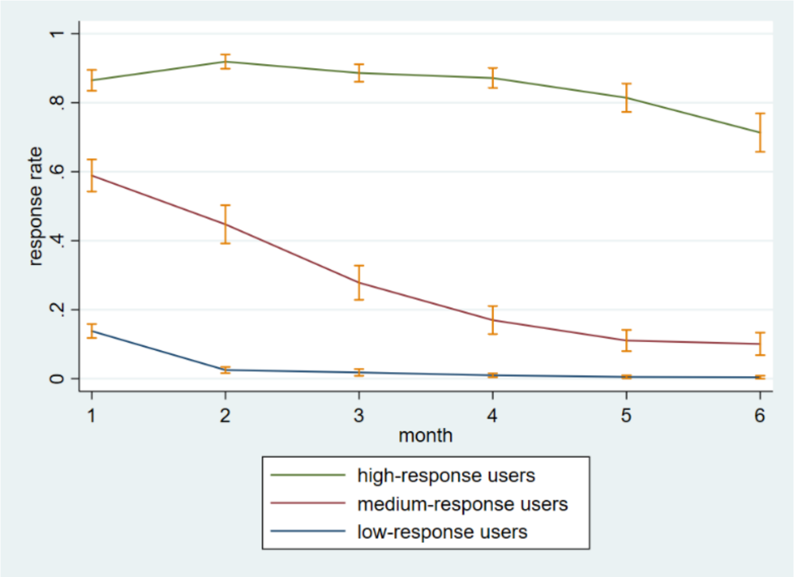

User groups, by level of 6-month response rate	Response rate per month, mean (95% confidence interval)					
	1	2	3	4	5	6
High:<br>> 0.6 & ≤ 1	0.86<br>(0.83, 0.90)	0.92<br>(0.90, 0.94)	0.89<br>(0.86, 0.91)	0.87<br>(0.84, 0.90)	0.81<br>(0.77, 0.86)	0.71<br>(0.66, 0.77)
Medium:<br>> 0.1 & ≤ 0.6	0.59<br>(0.54, 0.64)	0.45<br>(0.39, 0.50)	0.28<br>(0.23, 0.33)	0.17<br>(0.13, 0.21)	0.11<br>(0.08, 0.14)	0.10<br>(0.07, 0.13)
Low:<br>> 0 & ≤ 0.1	0.14<br>(0.12, 0.16)	0.02<br>(0.02, 0.03)	0.02<br>(0.01, 0.03)	0.01 (0.004, 0.02)	0.005<br>(0.001, 0.01)	0.004<br>(0.000, 0.01)

(b)

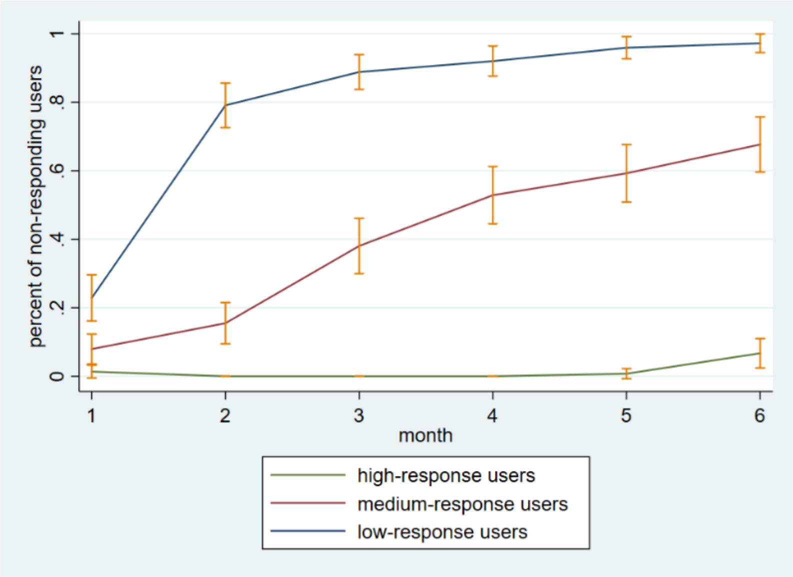

User groups, by level of 6-month response rate	Percent of users not responding (%)					
	1	2	3	4	5	6
High: > 0.6 & ≤ 1	0.01	0	0	0	0.01	0.07
Medium: > 0.1 & ≤ 0.6	0.08	0.15	0.38	0.53	0.60	0.68
Low: > 0 & ≤ 0.1	0.23	0.79	0.89	0.92	0.96	0.97
