# Supplementary material for: Evaluating the use of a recommender system for selecting optimal messages for smoking cessation: patterns and effects of user-system engagement
Source: BMC Public Health. 2021 Sep 26;21:1749. doi: 10.1186/s12889-021-11803-8 (PMC8465689; doi:10.1186/s12889-021-11803-8)
Supplement: Supplementary file 3 — Additional file 3. Impact of demographic factors on user-system engagement. [file 12889_2021_11803_MOESM3_ESM.pdf]

Additional File 3: Impact of demographic factors on user-system engagement

Table A3-1. User response rate and rating score of perceived influence of messages, stratified by demographic factors

	N_p (%)[a]	Response rate, mean (std)	*P* value[b]	N_r (%)[c]	Rating Score, mean (std)	*P* value
Total	731(100)			452 (100)		
Age group			0.004*			0.54
19-24 years	59 (8.1)	8.4 (15.2)[1]		30 (6.6)	4.4 (0.6)	
25-34 years	147 (20.1)	11.4 (17.2)[2]		95 (21.0)	4.2 (0.9)	
35-44 years	137 (18.7)	18.8 (21.8)[1,2]		93 (20.6)	4.2 (0.7)	
45-54 years	120 (16.4)	14.7 (21.0)		76 (16.8)	4.1 (0.8)	
55-64 years	207 (28.3)	13.1 (21.5)		125 (27.7)	4.1 (0.8)	
65+ years	61 (8.3)	9.8 (17.6)		33 (7.3)	4.1 (0.6)	
Gender			0.85			0.12
Female	531 (72.6)	13.5 (20.4)		331 (73.2)	4.1 (0.8)	
Male	200 (27.4)	13.2 (19.1)		121 (26.8)	4.2 (0.8)	
African-American			0.17			0.03*
Yes	97 (13.3)	16.0 (19.0)		62 (13.7)	4.3 (0.6)	
No	634 (86.7)	13.0 (20.2)		390 (86.3)	4.1 (0.8)	
Education			0.72			0.02*
≤High school	140 (28.2)	18.2 (21.5)		103 (28.3)	4.2 (0.6)[1]	
Some college or technical school	215 (43.3)	18.2 (22.1)		153 (42.0)	4.2 (0.8)[2]	
College graduate	141 (28.4)	20.0 (22.3)		108 (29.7)	4.0 (0.8)[1,2]	
How hard it is for you/family to pay for medical care			0.84			0.02*
Very hard	93 (18.7)	20.7 (22.0)		70 (19.2)	4.3 (0.6)	
Hard	75 (15.1)	17.6 (21.4)		58 (15.9)	4.0 (0.9)	
Somewhat hard	163 (32.8)	18.5 (22.8)		114 (31.2)	4.1 (0.7)	
Not very hard	153 (30.8)	18.6 (21.8)		113 (31.0)	4.1 (0.7)	
Don't know	13 (2.6)	14.8 (16.3)		10 (2.7)	4.7 (0.4)	
Number of cigarettes smoked per day			0.51			0.31
<=10	254 (34.7)	14.1 (19.8)		170 (37.6)	4.2 (0.8)	
>10 and <=20	331 (45.3)	12.5 (19.2)		199 (44.0)	4.2 (0.8)	
>20	146 (20.0)	14.4 (22.3)		83 (18.4)	4.0 (0.8)	

* indicates statistically significant (*P*<0.05).

[a] Demographic characteristics of 731 participants

[b] For each demographic factor, we used ANOVA to test its overall effects on the user response rate and the rating score of perceived influence respectively. For each polytomous factor that had an overall significant effect on the response rate or the rating score, we used the Bonferroni multiple-comparison test to assess the difference between each pair of categories. A pair with significant difference (*P*<0.05) is marked by the same superscript. Participants with age of 35-44 years had a higher response rate, compared with participants with age of 19-24 (18.8 vs. 8.4, *P*=0.01) or 25-34 (18.8 vs. 11.4, *P*=0.03) years. African American users assigned higher rating scores to the messages than non-African American users (4.3 vs. 4.1, *P*=0.03). College graduates assigned lower rating scores than participants with high school or lower-level education (4.0 vs. 4.2, *P*=0.03) and participants with some college or technical school education (4.0 vs. 4.2, *P*=0.04).

[c] Demographic characteristics of 452 participants who rated the messages
