# Supplementary material for: Evaluating the use of a recommender system for selecting optimal messages for smoking cessation: patterns and effects of user-system engagement
Source: BMC Public Health. 2021 Sep 26;21:1749. doi: 10.1186/s12889-021-11803-8 (PMC8465689; doi:10.1186/s12889-021-11803-8)
Supplement: Supplementary file 4 — Additional file 4. Top-10 high-impact messages sent by the recommender system. [file 12889_2021_11803_MOESM4_ESM.pdf]

Additional File 4: Top-10 high-impact messages sent by the recommender system

Message id	Message	Focuses of message content	Average rating score
EM2032[a]	Boredom can make your efforts to quit even harder. Make a list of easy and simple activities you can do when you get the urge to smoke. Start your list today and keep adding to it. Some ideas are: play a game of solitaire, walk around your work building once, do 10 jumping jacks, brush your teeth, file your fingernails, iron a shirt, play your favorite song, call a friend. For more ideas, visit the Decide2Quit website.	General treatment (Decide2Quit) and behavior treatment	4.682
EM3032	In five to 15 years of living smoke free, your risk of stroke goes down to a nonsmokers risk. Congratulations on a job well done!	Health benefits	4.667
EM1013	People who smoke often complain of feeling short of breath. Being short of breath from lack of oxygen can be a life-threatening condition and puts you at increased risk of stroke.	Health benefits	4.65
EM2036	Having cigarettes around may tempt you to smoke. Get rid of all cigarettes. Knowing you would have to go to the store just to have one will help you say "It's not worth it"!	Behavior treatment; motivation to quit	4.633
EM4003	You have done what many people struggle with daily-quit smoking. Don't you deserve a reward? Sometimes having a goal to focus on can help us stay motivated. If you are having trouble staying smoke free, set a goal for yourself of something really special you want to do. Save the money you used to spend on cigarettes to make it happen.	Behavior treatment; financial benefits	4.625
EM2034	Try different ways to distract yourself instead of smoking and see how which ones will work for you. Put something else in your mouth like a toothpick, piece of gum, a hard candy, lollypop, or a mint.	Behavior treatment	4.6
EM3010	Do you know how much money you are saving by quitting smoking? Each day you remain smoke free you are saving money!! Calculate your daily, weekly and monthly savings and plan to something special to reward yourself!	Financial benefits	4.6
EM2077	Realize that the first 48 hours is the most difficult time after quitting - and it gets better after that. Be ready with your strategies. Make a list ahead of time of how you will handle this challenge and plan for your success!	Behavior treatment	4.571
PM2Q30	Alan, a 64-year-old, thinks it is helpful to …<br><br>Remember why it is that you are quitting in the first place. Whether it be because of health or for your family, whatever, you need to keep your reason first and foremost in your thoughts.<br><br>You can build on your success by thinking about how well you've done in staying quit. Each time you resist, it will become a little easier.	Motivation to quit	4.569

EM2041	Try to see yourself at an event as a non-smoker. Be prepared by bringing things in your pocket or purse you know will keep you from smoking (hard candy, mint, toothpick, gum) and use these tools when you need them!	Behavior treatment	4.565

[a] EM: expert-created messages; PM: peer (i.e., smoker)-created messages
